# The Dual Role of Inducible Nitric Oxide Synthase in Myocardial Ischemia/Reperfusion Injury: Friend or Foe?

**DOI:** 10.1155/2018/8364848

**Published:** 2018-10-28

**Authors:** Xin Yu, Liang Ge, Liang Niu, Xin Lian, Haichun Ma, Lei Pang

**Affiliations:** ^1^Department of Hand Surgery, The First Hospital of Jilin University, Jilin, China; ^2^Department of Anesthesiology, The First Hospital of Jilin University, Jilin, China; ^3^Department of Operating Room, The First Hospital of Jilin University, Jilin, China; ^4^Department of Urology, The First Hospital of Jilin University, Jilin, China

## Abstract

Nitric oxide synthases (NOSs) are a family of enzymes that are responsible for the synthesis of nitric oxide (NO) from the amino acid L-arginine in the body. Among the three key NOSs, the expression of inducible NOS (iNOS) can only be induced by inflammatory stimuli and contribute to the large amount of NO production. iNOS-derived NO plays an important role in various physiological and pathophysiological conditions, including the ischemic heart disease. Nowadays, the development of specific iNOS inhibitors and the availability of iNOS knockout mice have provided substantial evidence to support the role of iNOS/NO signaling in the myocardium. Nevertheless, the role of iNOS/NO signaling in the myocardial ischemic reperfusion injury is very complex and highly perplexing; both detrimental and beneficial effects of iNOS have been described. Thus, this review will aim at providing basic insights into the current progress of the role of iNOS in myocardial ischemia reperfusion injury. A better understanding of the dual role of iNOS in details may help facilitate the development of more effective therapies for the management of ischemic heart diseases.

## 1. Introduction

Myocardial ischemic heart disease has been recognized as one of the main causes of death in the elderly in the industrialized world [1, 2]. It is characterized by insufficient blood supply to regions of the myocardium, which results in myocardial infarction, and further develops other disease states, such as hypertension, atherosclerosis, hyperlipidemia, diabetes, and heart failure. Timely reperfusion is one highly efficient treatment of this condition with mortality rate approximately half of hospitalized patients [3]. This procedure allows the rapid return of blood flow to the ischemic zone of the myocardium. However, reperfusion itself may lead to a consequence of tissue damage and pathological remodeling such as diminished cardiac contractile function, metabolic dysfunction, impairment of endothelial function, necrosis, and apoptosis [3]. All above complications further aggravate the degree of myocardial ischemia and eventually results in ischemia reperfusion injury [3, 4].

Nitric oxide (NO) is recognized as an important intracellular and intercellular biological active molecule that acts diverse physiological and pathophysiological functions in the body, including cardiac contractility and regulation of vasodilation [5]. However, the role of NO in myocardial damage and dysfunction during ischemia reperfusion remains controversial. The induction of inducible nitric oxide synthase (iNOS) produces excessive NO accompanied by increased production of reactive oxygen species (ROS), including peroxynitrite (OONO−) and superoxide, which are detrimental to the heart [6]. The expression of iNOS was also proved to correlate positively with the severity of cardiac dysfunction and expression of proinflammatory cytokines [7]. Nevertheless, endogenous NO production by NOSs may play a pivotal role for initiating and mediating the delayed role of ischemic preconditioning protection [8]. Clinical pretreatment with drugs, such as statins, certain calcium antagonists, angiotensin-converting enzyme (ACE) inhibitors, or dexamethasone, has been additionally reported to increase the release of NO and protect the myocardium against ischemia reperfusion injury [9]. Administration of NO or NO donors prior to ischemia also attenuates the consequences of myocardial ischemia reperfusion, including reduction of infarct size and endothelial dysfunction [10, 11]. Therefore, in this review, the focus will cover both damaging and protective effects of iNOS and its consequent NO production in myocardial ischemia reperfusion injury.

## 2. NO and NOS

NO is an inorganic free radical gas and a very small compound. Its function on vascular biology was discovered in the 1980s [12, 13]. In mammalian organism, NO is synthesized endogenously by converting L-arginine into L-citrulline. Overall oxidative reaction involves two separate mono-oxygenation steps that molecular oxygen utilizes NADPH as an electron donor and heme proteins, flavin mononucleotide (FMN), flavin adenine dinucleotide (FAD), and (6R-)5,6,7,8-tetrahydrobiopterin (BH4) as cofactors. NOSs are a family of enzymes that catalyze the production of NO from L-arginine in the body [14, 15]. There are three different isoforms of the NOS, which are referred to as neuronal NOS (nNOS or NOS I), inducible NOS (iNOS or NOS II), and endothelial NOS (eNOS or NOS III). Two enzymes, nNOS and eNOS, are also designated as constitutive NOS (cNOS) that generate and release NO mainly in resting cells, such as nerve cells and endothelial cells, thereby maintaining long-term regulation of synaptic transmission as well as the regulation of microvascular tone *in vivo*. In contrast to cNOS that can be activated by calcium and calmodulin (CaM) in healthy states, iNOS can only be induced by inflammatory stimuli including immunostimulatory cytokines, bacterial products, or infection in different types of cells, not in resting cells but in endothelium, monocytes, mast cells, macrophages, and smooth muscle cells [7, 15, 16].

Both nNOS and eNOS produce small amounts of NO (<100 nM) in the heart and are tightly controlled at posttranscriptional level [9, 17]. In response to myocardial ischemia, the level of cardiac nNOS is upregulated and inhibits xanthine oxidoreductase, leading to the inhibition of superoxide generation [18]. Additionally, L-type calcium current is also downregulated by nNOS overexpression, attenuating calcium overload due to the cardiac ischemic insult, thereby protecting the heart against ischemia reperfusion injury [19, 20]. The cardiac eNOS level is upregulated within minutes during ischemia, but the expression of eNOS is reduced with the prolonged cardiac ischemia [17]. Multiple lines of evidence signify the cardioprotective role of eNOS during myocardial ischemia reperfusion injury. Deficiency of eNOS in mice exhibited worse systolic and diastolic function and mortality after myocardial infarction when compared to wild type mice [21]. Moreover, cardiac-specific overexpression of eNOS in mice strongly protects against ischemia reperfusion injury *via* high levels of NO/cyclic guanosine monophosphate (cGMP) [22].

In contrast, iNOS produces larger amounts of NO (>1 *μ*M) and is primarily regulated at transcriptional level [9, 17]. The human iNOS gene is located at chromosome 17, around 37 kb with a high sequence similarity with nNOS and eNOS. The gene expression of iNOS and the subsequent mRNA translation are regulated by various stimuli, especially, lipopolysaccharide (LPS, endotoxin) and endogenous proinflammatory mediators, such as tumor necrosis factor-*α* (TNF-*α*), interleukin-1*β* (IL-1*β*), and interferon-*γ* (IFN-*γ*). Other stimulator, like hypoxia, is also demonstrated to be involved in the activation of iNOS gene expression [23]. One important intracellular signal transduction pathway of the above stimuli is the activation of NF-*κ*B signaling. In addition, the janus tyrosine kinase (JAK)–signal transducers and activators of transcription (STAT) together with the mitogen-activated protein kinases (MAPK) pathways are also involved in the contribution of iNOS gene expression. Posttranscriptional regulation of iNOS gene expression predominantly occurs *via* mechanisms that influence iNOS mRNA stability and regulation of catalytic activity [24].

### 2.1. The Detrimental Effect of iNOS/NO on Ischemia Reperfusion Injury

**Figure 1:**
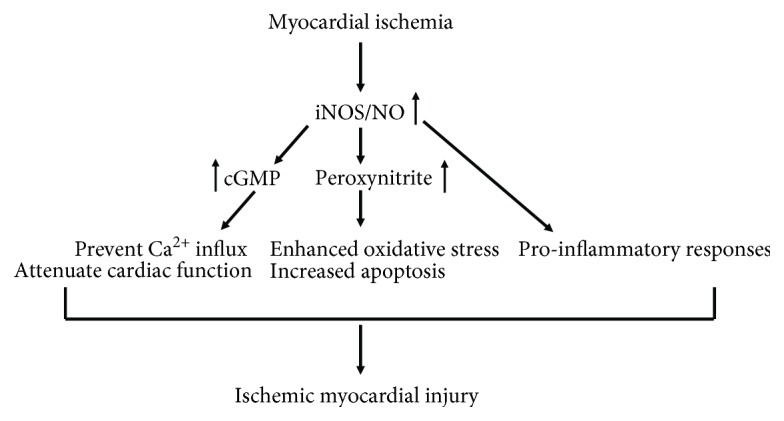
The detrimental effect of iNOS/NO on ischemia reperfusion injury. In response to myocardial ischemia, the upregulated iNOS-derived NO enhanced the level of intracellular cGMP, resulting in a decrease in Ca^2+^ influx, which depresses myofilament sensitivity to Ca^2+^ and subsequently attenuates cardiac contractile function. High levels of iNOS-derived NO also contribute to the formation of peroxynitrite, which subsequently leads to significantly increased oxidative stress and severe myocardial apoptosis. Together with iNOS/NO-mediated proinflammatory responses, these multiple actions of iNOS/NO exacerbate myocardial ischemia reperfusion injury.

NO could favor a detrimental role in myocardial ischemia reperfusion injury. Patel and his colleagues utilized in situ rabbit heart and demonstrated that pretreatment with an inhibitor of NO synthesis, L-NAME, significantly reduced infarct size following sustained coronary artery occlusion and reperfusion [25]. Mori's group later demonstrated that in dogs, NO production was enhanced in myocardial stunning and further aggravated cardiac damages through increased oxidative stress and the cytotoxicity. Such finding is the first time to report the detrimental role of NO in myocardial stunning *in vivo* [26]. During the reperfusion process, injury may occur in later stages associated with induction and activation of proinflammatory mediators, such as cytokines and iNOS [27, 28]. During the late phase of ischemia and reperfusion, high-output inducible iNOS/NO pathway was activated, which further aggravated left ventricular dysfunction and extent of myocardial infarct size. Whereas, continuous administration of aminoguanidine, a selective inhibitor of iNOS, can largely reduce infarct size and reverse myocardial injury [29]. Interestingly, during ischemia, overproduction of NO by upregulated iNOS for prolonged periods of time was suggested to act as a negative inotrope due to cardiac cGMP production [9, 30]. Increased iNOS activity also parallels the increment of intracellular cGMP through its subsequent production of NO. Of note, NO can prevent calcium (Ca^2+^) influx through cGMP-dependent inhibition of Ca^2+^ channels, which depresses myofilament sensitivity to Ca^2+^ and subsequently attenuates cardiac contractile function [31, 32]. Thus, enhanced cGMP level due to activated iNOS/NO signaling could be one of the underlying mechanisms that result in further myocardial injury during ischemia reperfusion **(**Figure 1**)**.

Besides the cGMP-dependent inhibition of Ca^2+^ channels, high levels of NO have also been proved to induce necrosis and apoptosis in cardiomyocytes after ischemia [33]. Under chronic isoproterenol stimulation, enhanced amount of NO induced by upregulated iNOS contributed to the formation of peroxynitrite, one byproduct of NO degradation, in the circulation system and heart, which subsequently leads to significantly severe myocardial apoptosis and eventually leads to an enlarged myocardial infarction size [34]. The formation of peroxynitrite induced by high levels of NO has been early demonstrated to be one of the important causes in myocardial damage [35]. The expression of iNOS stimulated elevations of NO and peroxynitrite formation in the myocardium of left ventricular and resulted in decrease myocardial function and less of survival, while iNOS knockout mice showed better myocardial contractility and higher survival rate when compared to wild type mice [35]. Zhu et al. reported that autophagy is additionally involved in the modulation of cell migration, and apoptosis in ischemia reperfusion mediated upregulation of iNOS [36]. Results suggested that ischemia reperfusion-induced iNOS-mediated nitrative stress increased the migration and apoptosis of human umbilical vein endothelial cells and further was associated with elevated autophagy, while the iNOS-specific inhibitor L-NAME attenuated cell apoptosis and migration induced by ischemia reperfusion [36]. Most recently, Jeddi et al. further showed evidence that the toxic effects of iNOS-derived NO during ischemia were due to increased peroxynitrite formation associated with enhanced apoptosis marker Bax/Bcl2 expression ratio in the cardiomyocytes, which results in myocardial injury [37]. Thus, enhanced peroxynitrite formation due to activated iNOS/NO signaling could contribute to the severe NO-induced myocardial apoptosis during ischemia reperfusion **(**Figure 1**)**.

Moreover, iNOS/NO signaling also plays a role in the modulation of the inflammatory responses caused by ischemia reperfusion [38]. Accumulating evidence indicated that the inflammatory response induces the release of inflammatory cytokines/chemokines which can recruit inflammatory cells like neutrophils and macrophages to migrate into the ischemic myocardium and produce more proinflammatory cytokines and chemokines, including TNF-*α*, IL-6, and MCP-1, which further exacerbate myocardial ischemia reperfusion injury [39]. Indeed, large amount of proinflammatory cytokines, such as IL-6 and TNF-*α*, are induced by stimulated iNOS after ischemia reperfusion, which lead to the heart tissue damage and extent ischemic myocardial injury and apoptosis [32, 40, 41] (Figure 1).

### 2.2. The Beneficial Effect of iNOS/NO on Ischemia Reperfusion Injury

In the past decade, a number of studies have focused on the function of NO in myocardial ischemia and its role in modulating the severity of ischemia reperfusion injury in non-preconditioned myocardium and proposed that iNOS/NO is harmful and aggravates myocardial ischemia reperfusion injury. However, overwhelming evidences have illustrated that NO overexpression by iNOS may actually have a protective role in mediating the antistunning and anti-infarct actions of ischemia-induced late preconditioning [42, 43].

Ischemic preconditioning is a well-described adaptive response of the heart that can protect against ischemia reperfusion injury through enhancing the ability to withstand a subsequent ischemic injury through a brief exposure to ischemic [44]. Study demonstrated that ischemic preconditioning upregulated iNOS expression in cardiomyocytes [45], and in conscious rabbits, the beneficial effects of late preconditioning against myocardial infarction were abrogated by the NOS inhibitor (N^w^-nitro-L-arginine) and iNOS inhibitors (aminoguanidine and S-methylisothiourea) [10, 46], suggesting that iNOS is an essential mediator of such cardioprotective responses. Moreover, late preconditioning can also be induced by NO donors, adenosine A (1) receptor agonists stimulation *via* an iNOS-dependent pathway in the isolated working heart from mice [47]. The lacking of protective effect of adenosine A (1) receptor activation in iNOS knockout mice additionally suggested a direct cause-and-effect relationship of iNOS in adenosine-induced late cardioprotection [8].

**Figure 2:**
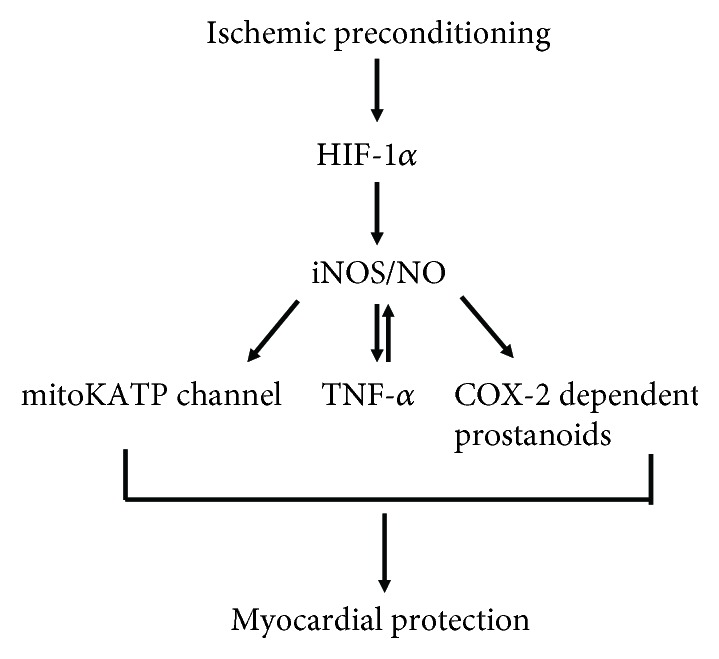
The beneficial effect of iNOS/NO on ischemia reperfusion injury. Enhanced iNOS-derived NO may be produced through HIF-1*α* signaling during ischemic preconditioning, resulting in the opening of mitoKATP channels and increased level of TNF-*α* and COX-2-dependent prostanoids, thereby mediating myocardial protection.

Whereas the mechanism whereby iNOS/NO during ischemic preconditioning protects the myocardium against ischemia reperfusion injury remains unclear, many hypotheses have been put forth. The opening of mitochondrial ATP-sensitive K^+^ (mitoKATP) channels has been demonstrated to play a significant role in delayed ischemic preconditioning (protection appears 24 h later) [48]. The usage of the mitoKATP channel opener diazoxide in mouse heart significantly activated iNOS and eNOS proteins through Akt/PI3 kinase signaling, which contributes the improvement of cardiac function and reduced apoptosis after ischemia reperfusion, and diazoxide was totally ineffective in iNOS knockout mice, suggesting that mitoKATP channel is an end effector of cardioprotection during delayed ischemic preconditioning [48, 49] (Figure 2). Furthermore, in wild type mice, ischemic preconditioning reduced myocardial infarction size, while in TNF-*α* knockout mice, such protective effects were abrogated [50, 51]. Similar results were also observed in TNF-*α* receptor knockout mice that the delayed cardioprotection against myocardial ischemia disappeared [52, 53]. TNF-*α* administration can also mimic the ischemic preconditioning and reduce infarction size, although the protective effects of TNF-*α* may only occur with low doses, but not higher doses [54]. Interestingly, iNOS and TNF-*α* have been demonstrated to be mutually regulated. NOS inhibition completely abolished the increased myocardial TNF-*α* levels secondary to coronary microembolization in a dog [55]. Exogenous TNF-*α* can enhance iNOS expression and subsequently lead to NO production in macrophage [56], and in transgenic mice with cardiac-specific overexpression of TNF-*α*, researchers demonstrated that the expression of iNOS as well as its activity was increased when compared to the control group [57]. Since myocardial ischemic reperfusion induced the increment of iNOS expression and subsequently stimulated TNF-*α* production, it is thus likely that iNOS/TNF-*α* signaling mediates cardioprotection against ischemic/reperfusion injury (Figure 2). However, further studies are still necessary to clarify this mechanism.

In addition, cyclooxygenase 2 (COX-2) has also been proved to be a mediator of iNOS-mediated cardioprotection during ischemic preconditioning [58]. COX-2 acts as a major player in inflammatory reactions and has been demonstrated to be highly expressed in the cardiac tissue during myocardial ischemia [59, 60]. Indeed, COX-2 and COX-2-dependent synthesis of prostanoids can mediate delayed preconditioning and play a role in cardioprotection [58]. In morphine-induced delayed cardioprotection model, the infarct-sparing effect after morphine administration was completely abolished by N-398 (COX-2-specific inhibitor). Furthermore, knockout of iNOS gene or administration of iNOS selective inhibitor (1400W) did not attenuate the increased expression of COX-2 protein after morphine pretreatment but completely abolished the upregulation of myocardial PGE_2_ and 6-keto PGF1*α*, indicating that iNOS is essential for the enzymatic activity of newly synthesized COX-2 following morphine pretreatment [58]. Taken together, these findings suggested that COX-2 may be located downstream of iNOS signaling in the protective pathway of delayed cardioprotection induced by morphine [58] (Figure 2).

During myocardial ischemia, the enhanced iNOS expression could be a consequence of activation of Hypoxia-inducible factor 1*α* (HIF-1*α*) signaling. HIF-1*α* is an important regulator in myocardial ischemia reperfusion injury in healthy hearts and can augment the purine signaling to facilitate myocardial protection [61]. Natarajan and colleagues showed the evidence that preserved HIF-1 can attenuate cardiac ischemia reperfusion injury through an iNOS-dependent preconditioning effect. Results demonstrated that increasing transcriptional activity of HIF-1*α* under normoxic conditions potently preconditions hearts against ischemic stress, although HIF-1*α* was active in hypoxic/ischemic states [62]. In addition, increasing HIF-1*α* transcriptional activity and iNOS mRNA expression by inhibition of HIF-1*α*-prolyl-4 hydroxylase-2 (PHD2) gene expression significantly reduced myocardial infarction size in mice with ischemia reperfusion, suggesting that the activation of HIF-1*α* in hearts by PHD2 siRNA administration attenuated reperfusion injury through an iNOS-dependent pathway [62]. Thus, enhanced cardiac iNOS may be produced through HIF-1*α* pathway during ischemic preconditioning (Figure 2).

## 3. Conclusion

Activation of iNOS/NO signaling exerts both protective and detrimental effects during myocardial ischemia reperfusion injury. The apparent controversy is possibly due to the critical balance between NO and peroxynitrite (one byproduct of NO degradation due to decreased NO bioavailability). In particular, iNOS-derived NO plays a cardioprotective role *via* its antioxidant and vasodilator effects in normal physiological conditions. However, in response to myocardial ischemia, enhanced iNOS/NO production led to the formation of peroxynitrite and its associated oxidative stress, which mediated the detrimental effects of iNOS/NO, whereas ischemic preconditioning markedly enhances the ability of the heart to withstand a subsequent ischemic injury and is closely associated with the upregulation of antioxidant defense system, which eliminates the increased oxidative stress [63]. In particular, the ischemic preconditioning suppresses the subsequent overproduction of peroxynitrite and protects the heart. Thus, the enhanced iNOS expression in conjunction with the elimination of oxidative stress switches iNOS from detrimental to protective NOS. In the future, with regard to the dual role of iNOS in myocardial ischemia reperfusion injury, a better understanding of iNOS/NO signaling is needed on how enhanced iNOS protects against ischemic heart disease without triggering unwanted side effects, with the aim to promote the development of more effective therapeutic approaches to treat ischemic heart diseases.
